# The positive role of Chinese herbal medicine as an adjunctive therapy for refractory gastroesophageal reflux disease: A systematic review and network meta-analysis

**DOI:** 10.1097/MD.0000000000042565

**Published:** 2025-05-23

**Authors:** Ruiting Zhang, Zhenyu Yang, Xiangbin Pan, Yingzhe Liu, Qiusi Huang, Zexi Song, Qianqian Yao, Dongxia Li, Yuan Zhang

**Affiliations:** a Heilongjiang University of Chinese Medicine, Harbin, China; b The Second Affiliated Hospital of Heilongjiang University of Chinese Medicine, Harbin, China; c The First Affiliated Hospital of Heilongjiang University of Chinese Medicine, Harbin, China.

**Keywords:** Chinese herbal medicine, network meta-analysis, pairwise meta-analysis, randomized controlled trials, refractory gastroesophageal reflux disease

## Abstract

**Background:**

Proton pump inhibitors are currently the primary treatment option for refractory gastroesophageal reflux disease (rGERD), yet they have limitations, including poor efficacy and potential adverse events. Chinese herbal medicine (CHM) may offer an effective and safe adjunctive therapy.

**Methods:**

This network meta-analysis investigate the adjunctive therapeutic effect and safety of CHM on rGERD. Randomized controlled trials (RCTs) of CHM combined with conventional Western medicine in the treatment of rGERD added to 8 online databases from its inception to January 2024 were systematically searched. Review Manager 5.3 and and Stata 14.0 software were used to conduct pairwise meta-analysis and network meta-analysis of RCTs that met the inclusion criteria. In addition, the methodological quality of RCTs was analyzed using the Cochrane Collaboration Risk of Bias ROB 2.0 assessment tool. This study was registered in PROSPERO (International Prospective Register of Systematic Reviews) (CRD42024532264).

**Results:**

This study included a total of 19 RCTs, comparing 16 CHMs. Pairwise meta-analysis indicated that CHM combined with conventional Western medicine outperformed the latter in terms of overall clinical efficacy, recurrence rate, and symptom improvement. Network meta-analysis suggested that Shugan Jieyu Capsule may significantly enhance overall clinical efficacy, while Buzhongyiqi Granules may significantly reduce the recurrence rate, Sanji Powders may significantly improve symptoms of acid reflux, Shugan Jieyu Capsule may significantly improve symptoms of heartburn, and Shugan Jianpi Hewei Decoction may significantly improve symptoms of esophageal chest pain, Qingweishu Granules may significantly improve the frequency scale for the symptoms of GERD score. No serious adverse events were reported in any of the RCTs.

**Conclusion:**

The findings of this study indicate that CHM offers a positive adjunctive therapeutic benefit for rGERD; however, it is imperative to enhance the quality of future RCTs to validate these preliminary findings.

## 1. Introduction

Gastroesophageal reflux disease (GERD) is a multifaceted disorder encompassing a family of syndromes attributable to, or exacerbated by gastroesophageal reflux.^[1]^ Heartburn, esophageal chest pain and regurgitation are the cardinal symptoms of GERD. Moreover, patients may also present with nausea, belching, abdominal pain and extra-esophageal symptoms such as chronic cough and wheezing.^[2]^ Epidemiological studies have shown that the global prevalence of GERD ranges from 7.4% to 19.6%, and its overall prevalence is increasing, especially in Asia.^[3,4]^ Proton pump inhibitors (PPIs) are the mainstay pharmacologic therapy for GERD. However, up to 40% of patients still exhibit GERD symptoms and objective evidence at least 8 weeks after optimizing the PPIs treatment plan, which known as refractory GERD (rGERD).^[5]^

Because of the high prevalence and chronic nature of rGERD, not only causes troublesome symptoms and complications to patients, which seriously affects the quality of life of patients, but also given its impact on consumption of healthcare resources. Currently, treatment for rGERD includes medication and invasive antireflux options. However, they have disadvantages such as lack of efficacy, adverse events, and surgical complications.^[6,7]^ Therefore, many patients turn to complementary and alternative therapies as a promising treatment option for rGERD, such as traditional Chinese medicine.^[8]^

Chinese herbal medicine (CHM) is an important part of traditional Chinese medicine, has been increasingly used in China, some other Asian countries and European countries.^[9]^ There have been a number of reports showing that CHM has a positive effect on improving the symptoms of patients with rGERD. It works primarily by inhibiting cellular inflammatory responses,^[10]^ antioxidant, regulating apoptosis and regulating hormone levels.^[11]^ In addition, CHM has the advantages of multicomponent, multi-target and multichannel,^[12]^ which can reduce the side effects and recurrence rate of conventional Western medicine, and even reduce the dose and course of conventional Western medicine, so the combination treatment of CHM and conventional Western medicine may be a new effective method. We conducted this study to provide more accurate evidence that CHM has a positive adjunctive therapeutic effect on rGERD, and to prioritize the clinical efficacy of different CHMs combined with conventional Western medicine in the treatment of rGERD, with a view to providing new ideas for the treatment dilemma of rGERD.

## 2. Materials and methods

This study was performed based on the preferred reporting item of the systematic review and meta-analysis.^[13]^ The PROSPERO (International Prospective Register of Systematic Reviews) number is CRD42024532264.

### 2.1. Search strategy

Two researchers independently screened the following databases from their inception to January 2024: PubMed, Web of Science, Cochrane, Embase, Chinese Biomedicine, China National Knowledge Infrastructure, Chinese Scientific Journals Database (VIP database for Chinese Technical Periodicals) and the WanFang Database. In addition, references included in the study were screened to expand the scope of the search. The key search words were: (Gastroesophageal reflux OR Gastric Acid Reflux OR Acid Reflux, Gastric OR Reflux, Gastric Acid OR Gastric Acid Reflux Disease OR Gastro-Esophageal Reflux Disease OR Gastro Esophageal Reflux Disease OR Gastro-Esophageal Reflux Diseases OR Reflux Disease, Gastro-Esophageal OR Gastro-esophageal Reflux OR Gastro-esophageal Reflux OR Reflux, Gastro-esophageal OR Gastroesophageal Reflux Disease OR GERD OR Reflux, Gastroesophageal OR Esophageal Reflux OR Gastro-Esophageal Reflux OR Gastro Esophageal Reflux OR Reflux, Gastro-Esophageal) AND (Refractory OR Persistent OR Troublesome OR Proton pump inhibit-resistant) AND (Chinese herb OR Herbal medicine OR Herbs OR Chinese medicine OR Chinese drug OR Traditional Chinese medicine OR Decoction OR Chinese Materia OR Tang OR Hangeshashinto OR Yukgunja-tang OR Rikkunshito OR Herbal formula).

### 2.2. Studies selection

The formulation of inclusion and exclusion criteria is based on the PICOS (participants, interventions, comparisons, outcomes and study design) principle.

Inclusion criteria: randomized controlled trials were conducted from their inception to January 2024; patients of any age or gender with a definitive diagnosis of rGERD; baseline comparability; the control group received conventional Western medicine treatment (such as PPIs, gastrokinetic drugs [GDL], etc), the experimental group was treated with CHM on the basis of the control group; the sample size of each trial should not be <20/arm; the duration of treatment is at least 4 weeks; outcome indicators include: overall clinical efficacy, recurrence rate, improvement of acid regurgitation, improvement of heartburn, improvement of esophageal chest pain, frequency scale for the symptoms of GERD (FSSG) score and adverse events.

Exclusion criteria: repeat published; non-RCTs; studies whose full text or complete data were not available after contacting the author; conference papers, reviews and theoretical discussions.

Any discrepancy was settled by a third person through negotiation and discussion.

### 2.3. Data extraction

Two investigators (Zhang and Yang) independently conducted data abstraction, including the following information: first author’s name, publication year, study country, sample size, patient age, duration, course of disease, intervention measure, outcomes, ingredients of CHM formula and adverse events. The primary outcome measure of this study was overall clinical efficacy, and the secondary outcome was recurrence rate, improvement of acid regurgitation, improvement of heartburn, improvement of esophageal chest pain, FSSG score and adverse events. Differences of opinion will be resolved by consultation among the members of the system evaluation team. If the data contained in the study is defective or missing, contact the first or corresponding author to seek data information.

### 2.4. Quality assessment

The quality of the included studies were analyzed using the Cochrane Collaboration Risk of Bias ROB 2.0 assessment tool, including selection bias (random sequence generation, allocation concealment); performance bias (blinding of participants and personnel); detection bias (blinding of outcome assessment); attrition bias (incomplete outcome data); reporting bias (selective reporting) and other bias. Two investigators (Zhang and Yang) evaluated the quality of the studies strictly according to the criteria and cross-checked the evaluation results.

### 2.5. Statistical analysis

Perform statistical analysis using Review Manager 5.3 and Stata 14.0 software. In terms of pairwise meta-analysis, relative risk (RR) with 95% confidence intervals (CIs) was used as the effect size of the statistical analysis, and the standardized mean difference (SMD) or mean difference of 95% CI was used to measure the statistical outcome of the data. The χ^2^ and *I*^2^ tests were used to assess heterogeneity. When the study heterogeneity was low (*P* > .1 or *I*^2^ ≤ 50%), the study was considered less heterogeneous. In this case, the fixed-effects model was adopted; otherwise, the random effect model was used. If heterogeneity is significant in the study, subgroup analysis and sensitivity analysis can be performed to explore possible causes of heterogeneity.

The network meta-analysis was conducted based on the frequency framework, and the relationship between various interventions was plotted as a network evidence map using Stata 14.0 software. Dichotomous variables were represented by odds ratio (OR), continuous variables by mean difference and each effect size was represented by 95% CI. In the network map, the larger the dots, the larger the sample size of the intervention, and the larger the lines, the more randomized controlled studies. For different outcome indicators, the surface under the cumulative ranking curve (SUCRA) was used to rank thetherapeutic effects of different interventions. The larger the SUCRA, the better the therapeutic effects of such interventions. Drawing comparation-corrected funnel plots to assess publication bias in included studies; when there is a closedloop, an inconsistency check is performed.

## 3. Results

### 3.1. Characteristics of included studies

**Figure 1.**
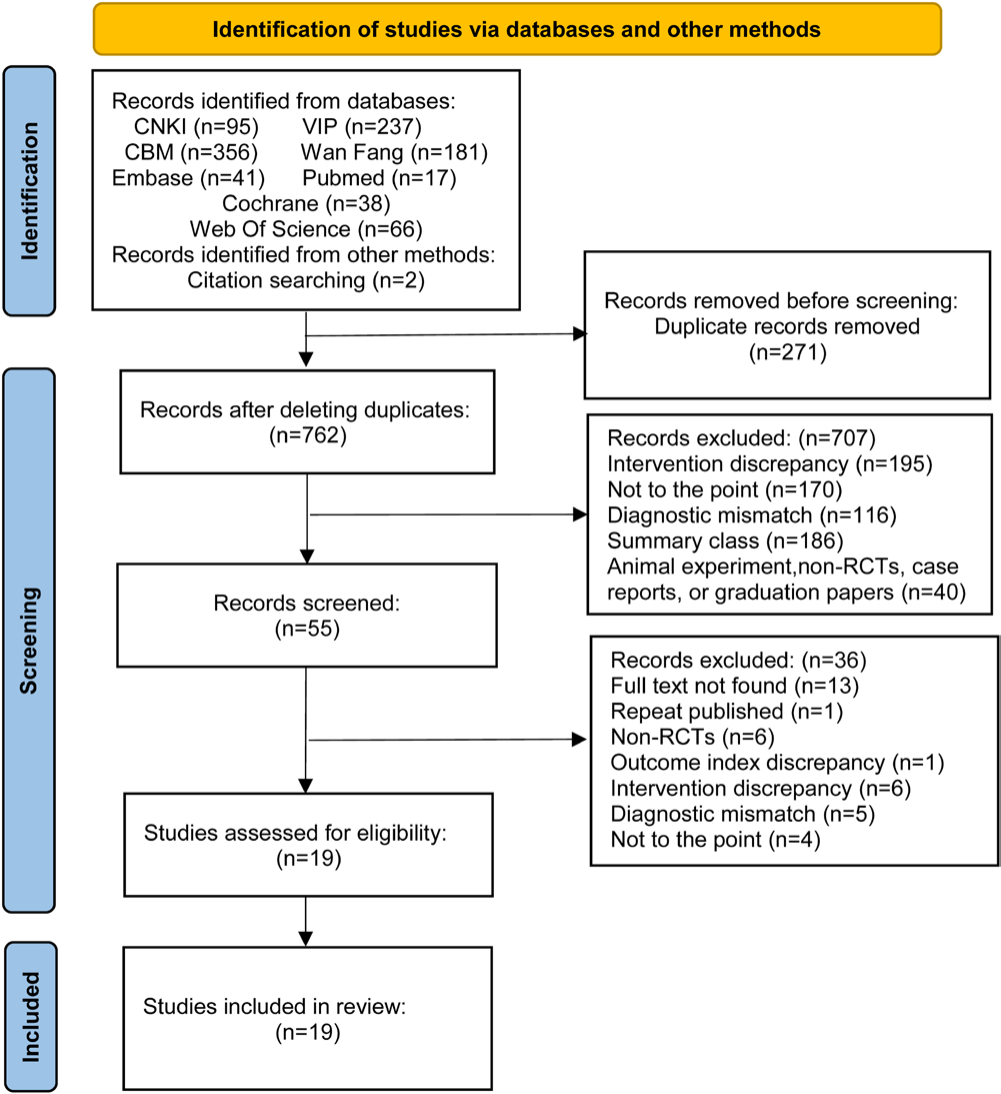
Literature screening process. CBM = China Biology Medicine database, CNKI = China National Knowledge Infrastructure, non-RCTs = non-randomized controlled trials, VIP = VIP database for Chinese Technical Periodicals.

A total of 1033 studies were screened from 8 databases. After reading the title and abstract, 978 studies were excluded due to duplication and irrelevance. Then, the remaining 55 studies were screened for full text. Based on established inclusion and exclusion criteria, 36 studies were excluded. Finally, 19 studies were included in this study, including 4 published in English^[14–17]^ and 15 published in Chinese.^[18–32]^ The specific literature screening process is shown in Figure 1.

Table 1 summarizes the baseline characteristics of 19 studies. Four studies were multicenter studies and the remaining 15 were single-center studies. A total of 1585 patients were enrolled, including 728 males and 847 females. Sample sizes ranged from 40 to 217. The studies were published between 2011 and 2023. Included patients ranged in age from 20 to 90 years old; the course of disease ranged from 2.8 months to 30 years. The course of treatment was 4 to 12 weeks. The control group of all studies were treated with conventional Western medicine, such as PPIs, GDL, etc.

**Table 1:** Characteristics of the studies included in the network analysis.

Author year	Country	Simple size (male/female)	Duration (wk)	Age (y)	Course of disease (months)	Intervention		Outcomes	Ingredients of HM formula
						EG	CG		
Tominaga 2014^[14]^	Japan	E: 109 (35/74) C: 108 (28/80)	No. 4, 8	E: 62.1 (25–85) C:59.4 (22–83)	N/A	Rikkunshito + CT	CT	② ③ ㉕ ㉖	Atractylodes lancea Rhizome, Ginseng, Pinellia tuber, Poria sclerotium, Jujube, Citrus unshiu Peel, Glycyrrhiza, Ginger
Tominaga 2012^[15]^	Japan	E: 48 (20/28) C: 51 (17/34)	4	E: 63.6 (25–86) C: 64.5 (25–90)	N/A	Rikkunshito + CT	CT	② ㉖	Atractylodes lancea Rhizome, Ginseng, Pinellia tuber, Poria sclerotium, Jujube, Citrus unshiu Peel, Glycyrrhiza, Ginger
Takeuchi 2019^[16]^	Japan	E: 38 (10/28) C: 32 (11/21)	4	E: 59.2 ± 14.7 C: 60.8 ± 14.7	N/A	Hangeshashinto + CT	CT	② ㉖	Pinellia tuber, Jujube, Ginseng, Processed ginger, Coptis rhizome, Scutellaria root, Glycyrrhiza
Sakata 2014^[17]^	Japan	E: 52 (17/35) C: 43 (8/35)	No.4, 8	E: 72.1 (65–85) C: 73.4 (65–83)	N/A	Rikkunshito + CT	CT	② ㉕	Atractylodes lancea Rhizome, Ginseng, Pinellia tuber, Poria sclerotium, Jujube, Citrus unshiu Peel, Glycyrrhiza, Ginger
Shen 2023^[18]^	China	E: 40 (24/16) C: 40 (27/13)	8	E: 56.12 ± 5.49 C: 55.68 ± 5.53	E: 16.80 ± 4.02 C: 16.34 ± 3.95	SGHWAS + CT	CT	④ ⑤ ⑥ ⑦	Albiziae cortex, Endoconcha sepiae, Aurantii fructus, Paeoniae radix alba, Atractylodes macrocephala Koidzumi, Cyperi Rhizoma, Polygalae Radix, Citri Reticulatae Pericarpium, Bupleurum Radix, Coptwas chinenswas Franch, Evodia rutaecarpa
Yu 2017^[19]^	China	E: 38 (28/10) C: 35 (24/11)	8	E: 48.71 ± 10.18 C: 47.62 ± 9.74	E: 30.13 ± 12.22 C: 28.62 ± 12.56	BXHP + CT	CT	① ⑧ ⑨	Pinellia tuber, Magnolia cortex, Poria, Perilla leaf, Ginger rhizome
Yao 2022^[20]^	China	E: 42 (18/24) C: 42 (20/22)	4	E: 42.19 ± 8.25 C: 42.31 ± 8.57	N/A	DXZY + CT	CT	④ ⑩ ⑪ ㉖	Pericarpium citri Reticulatae, Bupleurum chinense, Glycyrrhiza uralensis Fisch, Codonopsis pilosula, Evodia rutaecarpa, Pinellia tuber, Atractylodes macrocephala, Syzygium aromaticum, Phellodendron, Cimicifugae Rhizoma, Astragali Radix, Angelicae Sinensis Radix, Alpinia katsumadai
Xie 2021^[21]^	China	E: 41 (19/22) C: 41 (17/24)	12	E: 47.21 ± 8.91 C: 48.01 ± 9.08	E: 15.78 ± 3.63 C: 16.05 ± 3.75	SGJY + CT	CT	① ⑩ ⑫ ⑬ ⑭	Hypericum perforatum, Acanthopanax senticosu
Liu 2018^[22]^	China	E: 60 (24/36) C: 60 (32/28)	8	E: 47.55 ± 12.54 C: 46.03 ± 11.36	E: 18 ± 192 C: 30 ± 192	QW + CT	CT	① ② ④ ㉒ ㉖	Inula japonica Thunb, Haematitum, Paeonia lactiflora, Salvia miltiorrhiza, Cynanchum paniculatum, Corydalis yanhusuo, Cyperi rhizoma, Forsythiae Fructus
Cao 2020^[23]^	China	E: 30 (14/16) C: 30 (15/15)	4	E: 43.23 ± 10.55 C: 42.33 ± 9.5	E: 24.97 ± 11.52 C: 25.37 ± 13.89	HG + CT	CT	① ④ ⑧	Gardenia jasminoides Ellis, Pericarpium citri Reticulatae, Citri Reticulatae Pericarpium Viride, Perillae caulis, Poria, Paeonia lactiflora, Radix Aucklandiae, Fossilia Ossis Mastodi, Fritillariae thunbergii bulbus, Fingered citron, Rice-grain Sprout
Zheng 2018^[24]^	China	E: 30 (11/19) C: 30 (12/18)	8	E: 55.5 ± 6.5 C: 55.6 ± 6.4	E: 3.6 ± 0.8 C: 3.8 ± 0.6	SJ + CT	CT	① ⑪ ⑮ ㉒ ㉖	Panax notoginseng, Bletilla striata
Qi 2023^[25]^	China	E: 38 (16/22) C: 37 (16/21)	No. 2, 4	E: 53.95 ± 10.72 C: 57.41 ± 9.76	N/A	SGHW + CT	CT	⑩ ⑯ ⑰ ⑱ ㉖	Gardeniae Fructus, Magnolia cortex, Bupleurum Radix, Coptwas chinenswas Franch, Evodia rutaecarpa, Inula japonica Thunb, Calm shell, Corydalis yanhusuo, Ginger, Fructus Aurantii, Polygonatum sibiricum, Cyperi Rhizoma
Que 2017^[26]^	China	E: 30 (19/11) C: 34 (21/13)	8	E: 23–69 C: 22–69	N/A	SGHW + CT	CT	① ④ ⑪ ⑭ ⑲ ㉖	Gardeniae Fructus, Magnolia cortex, Bupleurum Radix, Coptwas chinenswas Franch, Evodia rutaecarpa, Inula japonica Thunb, Calm shell, Corydalis yanhusuo, Ginger, Fructus Aurantii, Polygonatum sibiricum, Cyperi Rhizoma
Li 2016^[27]^	China	40 (22/18)	12	20–73	N/A	SGJPHW + CT	CT	① ⑰	Bupleurum Radix, Coptwas chinenswas Franch, Evodia rutaecarpa, Inula japonica Thunb, Fructus Aurantii, Cyperi Rhizoma, Citri Reticulatae Pericarpium, Paeoniae Radix Alba, Poria, Perilla leaf, Atractylodes macrocephala Koidzumi, Haematitum, Pinellia tuber, Glycyrrhiza, Radix Codonopsis, Green Tangerine Peel
Huang 2022^[28]^	China	E: 34 (20/14) C: 34 (19/15)	8	E: 51.48 ± 6.42 C: 52.12 ± 6.81	E: 36.24 ± 13.32 C: 38.04 ± 13.92	ZZHP + CT	CT	① ④ ⑳ ㉑ ㉖	Gardeniae Fructus, Magnolia cortex, Glycyrrhiza, Radix Codonopsis, Coptwas chinenswas Franch, Angelica sinensis, Aurantii fructus immaturus, Sojae Semen Praeparatum, Mume Fructus，Dried Ginger，Cinnamomi Ramulus，Phellodendron
Deng 2018^[29]^	China	60 (33/27)	8	49.44 ± 4.87	N/A	BZYQ + CT	CT	㉒ ㉓ ㉔	Astragalus mongholicus Bunge, Glycyrrhiza uralensis Fisch, Codonopsis pilosula, Angelica sinensis, Pericarpium citri Reticulatae, Bupleurum chinense, Atractylodes macrocephala Koidz, Rhizoma Cimicifugae Foetidae, Ginger rhizome, Jujube
Liang 2023^[30]^	China	E: 44 (23/21) C: 44 (25/19)	8	E: 49.34 ± 9.60 C: 48.18 ± 10.60	E: 49.92 ± 30.12 C: 45.6 ± 28.2	JPSG + CT	CT	① ④ ⑧ ⑬ ㉒ ⑪㉖	Pericarpium citri Reticulatae, Bupleurum chinense, Atractylodes macrocephala Koidz, Codonopsis pilosula, Pinellia tuber, Chuanxiong Rhizoma, Glycyrrhiza uralensis Fisch, Fructus Aurantii, Cyperi Rhizoma, Poria, Paeonia lactiflora, Clam shell, Plum blossom
Fan 2022^[31]^	China	E: 50 (28/22) C: 50 (26/24)	8	E: 54.64 ± 10.17 C: 51.26 ± 10.68	E: 69.12 ± 44.28 C: 68.64 ± 55.8	QWS + CT	CT	① ⑭ ⑲ ㉒	Coptwas chinenswas Franch, Fritillariae Thunbergh Bulbus, Pinellia tuber, Citri Reticulatae Pericarpium, Endoconcha sepiae, Caulis Bambusae in Taenia, Pericarpium Trichosanthes, Fennel, Dandelion, Panax notoginseng, Amomi fructus, Arca Concha
Zhao 2022^[32]^	China	E: 25 (15/10) C: 25 (14/11)	8	E: 39.4 ± 5.6 C: 38.8 ± 5.5	E: 33.6 ± 14.4 C: 34.8 ± 16.8	ECPW + CT	CT	① ⑩	Pericarpium citri Reticulatae, Atractylodes macrocephala Koidz, Pinellia tuber, Glycyrrhiza uralensis Fisch, Fructus Aurantii, Poria, Inula japonica Thunb, Haematitum, Evodia rutaecarpa, Bupleurum chinense, Cyperi Rhizoma, Coptwas chinenswas Franch, Magnolia officinalis, Atractylodis Rhizoma

Outcomes: ① Overall clinical efficacy. ② Frequency Scale for the Symptoms of Gastroesophageal Reflux Disease (FSSG). ③ Short-Form Health Survey-8 (SF-8). ④ TCM syndrome integral. ⑤ The level of esophageal dynamic function. ⑥ Gastrointestinal hormone levels. ⑦ Sleep quality. ⑧ GERD Questionnaire scores (GerdQ). ⑨ Pressure of lower esophageal sphincter (LES). ⑩ Reflux Disease Questionnaire (RDQ). ⑪Scores of gastroscopy evaluation. ⑫ Hamilton depression scale(HAMD). ⑬ Hamilton anxiety scale (HAMA). ⑭ Short-Form Health Survey-36 (SF-36). ⑮ Reflux esophageal inflammatory integrals. ⑯ GERD Health-Related Quality of Life Questionnaire (GERD-HRQL). ⑰ Total clinical symptoms score. ⑱ Self-rating scale of somatization symptoms (SSS). ⑲ SDS and SAS scales. ⑳ RDQ Symptom frequency integral.㉑ RDQ Symptom severity score. ㉒ Recurrence rate. ㉓ Symptom score (according to heartburn, acid reflux, belching, retrosternal pain, nausea, vomiting). ㉔ Curative effect under gastroscopy. ㉕ Gastrointestinal Symptom Rating Scale (GSRS). ㉖ Adverse events.

BXHP = Banxia Houpu Decoction, BZYQ = Buzhongyiqi Granules, C = control group, CT = conventional Western medicine, DXZY = Dingxiang Zhuyu Decoction, E = experimental group, ECPW = Erchen Pingwei Power, HG = Huagan Decoction, JPSG = Jianpi Shugan Decoction, N/A = not applicable, QW = Qingwei Granules, QWS = Qingweishu Granules, SGHW = Shugan Hewei Decoction, SGHWAS = Shugan Hewei Anshen Prescription, SGJPHW = Shugan Jianpi Hewei Decoction, SGJY = Shugan Jieyu Capsule, SJ = Sanji Powders, ZZHP = Zhizi Houpo Decoction and Zhizi Douchi Decoction Combined with Wumei Pill.

Various types of Chinese herbal decoction including Rikkunshito (3), Shugan Hewei Decoction (2), Hangeshashinto (1), Banxia Houpu Decoction (1), Shugan Hewei Anshen Prescription (1), Dingxiang Zhuyu Decoction (1), Shugan Jieyu Capsule (1), Qingwei Granules (1), Huagan Decoction (1), Sanji Powders (1), Shugan Jianpi Hewei Decoction (1), Zhizi Houpo Decoction and Zhizi Douchi Decoction Combined with Wumei Pill (1), Buzhongyiqi Granules (1), Jianpi Shugan Decoction (1), Qingweishu Granules (1), Erchen Pingwei Power (1) were utilized combine with conventional Western medicine in the experimental group. The control group was treated with conventional Western medicine, such as PPIs, GDL, etc. In addition, in order to better understand relevant herbs, we searched and displayed the sources and main indications of herbs involved in 19 studies, involving a total of 62 herbs (see Table 2 for details).

**Table 2:** Sources and main indications of herbal medicines.

Herbal name	Source	Main indications
Atractylodes lancea Rhizome	It is the rhizome of *Atractylodes australis* or *Atractylodes borealis* of Compositaceae	Wet sheng trapped spleen, lethargy, abdominal distension, loss of appetite, vomiting, diarrhea, dysentery, malaria, phlegm, edema, when qi cold, wind cold wet arthralgia, foot flaccidity, night blindness
Ginseng	The root of ginseng, a plant of the Panax genus in the Araliaceae family	Treat strain injury and deficiency, food deficiency, fatigue, nausea and vomiting, stool slip, vacuous cough and wheezing, spontaneous sweating, palpitation, forgetfulness, vertigo, headache, impotence, frequent urination, thirst dissipation, women’s leakage, children’s slow terror, and long deficiency, all deficiency of qi and blood body fluid
Pinellia tuber	It is a tuber of pinellia in the araceae family	Excessive sputum cough and panting, sputum and drink dizziness palpitation, internal sputum dizziness, vomiting and nausea, chest duct fullness and depression, plum kernel gas disease
Poria sclerotium	It is the dried sclerotium of poraceae fungus Poria	Urine adverse, water swelling full, phlegm drink cough, vomiting, diarrhea, spermatospermia, opacity, palpitations, forgetfulness
Jujube	It is the dried and mature fruit of jujube in rhamnus family	Lack of stomach and food, weak spleen and loose stool, insufficient qi and blood body fluid, Ying Wei disharmony, palpitation and palpitation. The woman is restless
Glycyrrhiza	It is the root and rhizomes of licorice legumes	Weakness of the spleen and stomach, lack of food, abdominal pain and loose stool, fatigue and fever, lung flaccidity and cough, palpitation, convulsive epilepsy; For the treatment of sore throat, peptic ulcers, boils, antidotes and food poisoning
Ginger	It is the fresh rhizome of ginger	Cold wind cold, vomiting, phlegm, wheezing cough, fullness, diarrhea, detoxification
Coptis rhizome	The dried rhizome of *Coptis chinensis* in the goldenseal family	Damp-heat full, vomiting and acid swallowing, diarrhea, jaundice, high heat god faint, heart fire hyperactivity, upset insomnia, blood heat epistaxis, red eyes, toothache, thirst, carbuncle and pustule; External treatment of eczema, wet sores, ear canal discharge pus
Scutellaria root	It is the root of *Scutellaria scutellaria* in the labiaceae family	Strong heat and thirst, lung heat cough, damp-heat diarrhea, jaundice, heat, vomiting, epistaxis, insipidus, leakage, eye red swelling pain, restless fetus, swollen uncle
Albiziae cortex	The dried bark of the legume albizia	Uneasiness, melancholy insomnia, lung carbuncle, sore, fall pain
Endoconcha sepiae	It is a dried inner shell of *Sepiella maindronide* Rochebrune or *Sepia esculenta* Hoyle	Stomachache swallowing acid, vomiting, epistaxis, hematemesis, hematochezia, bleeding under the leaky belt, blood dry amenorrhea, abdominal pain syndrome, deficiency malaria diarrhea, Yin erosion and sore
Aurantii fructus	Dried young fruits of sour or sweet oranges in the Rutaceae family	Chest and abdomen fullness, chest obstruction, pain, phlegm addiction, edema, food accumulation, constipation, gastroptosis, uterine ptosis, anal prolapse
Paeoniae radix alba	Dried roots of peony, a plant in the Ranunculaceae family	Chest, abdominal, and rib pain, diarrhea and abdominal pain, spontaneous sweating and night sweats, yin deficiency and fever, menstrual disorders, diarrhea and diarrhea, vaginal discharge
Atractylodes macrocephala Koidzumi	It is the dried rhizome of *Atractylodes macrocephala* koidz	The spleen and stomach qi is weak, does not think of diet, is tired and has little Qi, empty distension, diarrhea, phlegm, edema, jaundice, dampness, urination is unfavorable, dizziness, spontaneous sweating, fetal gas is restless
Cyperi Rhizoma	It is the dried rhizome of *Cyperus rotundus* L	Swelling pain in the chest, abdomen and hypochondrium, sputum and drink fullness, irregular menstruation, discontinuous leakage.
Polygalae Radix	It is the root of *Polygala tenuifolia*	Insomnia and dreaminess, forgetfulness and palpitations, trance, uncomfortable expectoration, sores, swelling and toxin, breast swelling and pain
Citri Reticulatae Pericarpium	Dried and mature peel of *Citrus reticulata* Blanco and its cultivated varieties in Rutaceae	Full chest and abdomen, no appetite, vomiting, cough and phlegm
Bupleurum Radix	It is the dried root of *Bupleurum chinense* or *Bupleurum angustifoliae*	Exchange of cold and heat, chest full of flank pain, bitter mouth and deafness, headache and dizziness, malaria, anal prolapse, irregular menstruation, uterine prolapse
Evodia rutaecarpa	It is the immature fruit of Rutaceae plants *Evodia rutaecarpa*, stone tiger and Evodia pilosa	Vomiting and acid swallowing, Jueyin headache, visceral cold vomiting and diarrhea, abdominal distension and pain, menstrual abdominal pain, diarrhea at the fifth Watch
Magnolia cortex	Dry bark, root bark, and branch bark of Magnoliaceae plant Magnolia officinalis	Chest and abdomen fullness of distending pain, nausea, vomiting, overnight food does not go away, sputum and drink wheezing cough, cold and damp diarrhea
Perilla leaf	Dried leaves of perilla, a plant in the family Lamiaceae	Cold, fever, cough, wheezing, chest and abdominal distension, restless fetus. And can detoxify fish and crab
Codonopsis pilosula	Dried roots of *Codonopsis pilosula*, a plant in the Campanulaceae family	Weakness of the spleen and stomach, deficiency of qi and blood, fatigue and weakness of the body, insufficient appetite and loose stools, internal heat to quench thirst, prolonged diarrhea, prolapse, lung deficiency, wheezing and coughing, shortness of breath and self sweating, and slight deficiency of qi
Syzygium aromaticum	Dried flower buds of *Eugenia caryophllata* Thunb in the family Myrtle	Hiccup, cold and painful epigastric pain, vomiting and diarrhea due to insufficient food intake, kidney deficiency and erectile dysfunction, soreness and coldness in the waist and knees, and Yin gangrene
Phellodendron	Dry bark of the Rutaceae plant Phellodendron or Phellodendron bark	Hot diarrhea, diarrhea, thirst quenching, jaundice, nocturnal emission, gonorrhea, hemorrhoids, bloody stools, red and swollen eyes, mouth and tongue sores, and swollen and toxic sores
Cimicifugae Rhizoma	The rhizomes of the Ranunculaceae plant Cistanche	Epidemic, fever, headache, sore throat, mouth sores, and incomplete rash; Qi sinking, prolonged diarrhea and dysentery, prolapse of the anus, vaginal discharge, uterine prolapse
Astragali Radix	The root of *Astragalus membranaceus*, a plant of the genus Astragalus in the legume family	Qi deficiency and weakness, insufficient appetite, loose stools, and sinking of qi
Angelicae Sinensis Radix	Dried roots of Angelica sinensis, a plant in the Umbelliferae family	Abdominal pain due to amenorrhea, clustering of symptoms, collapse, blood deficiency, headache, dizziness, etc
Alpinia katsumadai	The rhizomes of the ginger plant, Galangal, in the ginger family	Cold in the spleen and stomach, cold and painful in the epigastric region, vomiting and diarrhea, choking and nausea, and food stagnation
Hypericum perforatum	The whole plant or rooted whole plant of *Forsythia suspensa* in the family Theaceae	Hemoptysis, vomiting blood, traumatic bleeding, rheumatism and bone pain, mouth and nose sores, swelling and toxin, soup fire injury
Acanthopanax senticosu	Dry roots, rhizomes, or stems of *Acanthopanax senticosus* plants in the Araliaceae family	Weakness and fatigue, loss of appetite, soreness in the waist and knees, insomnia and frequent dreams
Inula japonica Thunb	The inflorescence of the spiral flower or Eurasian spiral flower in the Asteraceae family	Swelling under the lower abdomen, coughing and wheezing, hiccups, hardness of the chest, inability to expel qi, and swelling of the upper abdomen
Haematitum	Dried roots of Paeonia lactiflora Pall or *Paeonia veitchii* Lynch in the Ranunculaceae family	Stagnation of meridians, accumulation of hernias, abdominal pain, rib pain, bleeding, dysentery, intestinal bleeding under the wind, redness of the eyes, and abscess
Salvia miltiorrhiza	It is the root of *Salvia miltiorrhiza* in the labiaceae family	Angina pectoris, menstrual disorders, dysmenorrhea, amenorrhea, bleeding under the belt, joint pain, palpitations and insomnia, and malignant sores and swelling
Cynanchum paniculatum	The roots, rhizomes, or whole plants with roots of the medicinal plant Xu Changqing in the family Asclepiadaceae, a dicotyledonous plant	Stomachache, toothache, rheumatic pain, menstrual abdominal pain, chronic tracheitis, ascus, edema, dysentery, enteritis, bruising injury, eczema, urticaria, snakebite
Corydalis yanhusuo	Tubers of the poppy plant *Corydalis yanhusuo*	Pain in the heart, abdomen, waist, and knees, irregular menstruation, postpartum dizziness, persistent lochia, and injuries from falls and injuries
Forsythiae Fructus	Dried fruit of *Forsythia suspensa*, a plant in the Oleaceae family	Warm-heat disease, erysipelas, rashes, abscesses and swelling, scrofula, and urinary obstruction
Gardenia jasminoides Ellis	Dried and Mature Fruit of *Gardenia jasminoide*s, a Plant in the Rubiaceae Family	Sleepless due to restlessness, jaundice, gonorrhea, consumptive thirst, red eyes, sore throat, vomiting blood, bleeding, bloody dysentery, hematuria, heat toxin sores, sprains, swelling and pain
Pericarpium citri reticulatae viride	Peel of young or immature fruits of the Rutaceae plant orange and its cultivated varieties	Chest, rib, and epigastric pain, hernia, food accumulation, breast swelling, breast nucleus, and persistent malaria mass
Radix Aucklandiae	The root of the Auckiandialappa Decne in the Asteraceae family	Middle cold qi stagnation, chest and abdominal distension and pain, vomiting, diarrhea, dysentery, cold hernia
Fossilia Ossis Mastodi	Fossils of bones from ancient large mammals such as elephants, 3 toed horses, rhinoceroses, deer, cows, etc	Startled epilepsy, palpitations and forgetfulness, insomnia and frequent dreams, self sweating and night sweats, nocturnal emission and turbidity, vomiting and bleeding in the stool, diarrhea and prolapse, ulcers that persist for a long time
Fritillariae thunbergii bulbus	The bulbs of the lily family plant *Fritillaria thunbergii*	Wind heat cough, lung abscess and throat obstruction, scrofula, sores, swelling and toxin
Fingered citron	The mature fruit of Citrus or Fingered citron, a plant in the Rutaceae family	Stomach pain and bloating, phlegm and cough, vomiting and eating less
Rice-grain Sprout	Mature fruit of rice, a grass plant	Unable to digest overnight food, bloating, diarrhea, and lack of appetite
Panax notoginseng	The root tuber of Panax notoginseng, a plant in the Panax genus of the Araliaceae family	Vomiting blood, coughing up blood, bleeding, rectal bleeding, bloody dysentery, postpartum dizziness, traumatic bleeding, swelling and pain
Bletilla striata	Dried tubers of the orchid plant Bai Ji	Coughing up and vomiting blood, bleeding from external injuries, burns from soup and fire, ulcers and swelling, pulmonary tuberculosis coughing up blood, and bleeding from ulcer disease
Arca Concha	The shells of Arca subcrinata Lischke, Arca granosa Linnaeus, or Arca inflata Reeve, which belong to the family of clams	Phlegm accumulation, stomach pain, noise, acid regurgitation, scrofula
Fructus Aurantii	Dried immature fruits of *Citrus aurantium* L and its cultivated varieties in the Rutaceae family	Chest distension, bloating, food accumulation, belching, nausea and vomiting, diarrhea, prolapse of the anus, and uterine prolapse
Polygonatum sibiricum	The rhizomes of plants such as *Polygonatum sibiricum* and *Polygonatum sibiricum* in the lily family	Yin deficiency and cough, lung dryness and cough, spleen deficiency and fatigue, lack of food and dry mouth, consumptive thirst, kidney deficiency and soreness in the waist and knees, impotence and nocturnal emission
Caulis Perillae	Wrinkle the stems of plants in the family Lamiaceae, such as *Perilla frutescens* and *Perilla frutescens*	Qi stagnation, food stagnation, chest and diaphragm tightness, abdominal pain, fetal qi imbalance
Hematite	Ore of oxide mineral hematite	Ejaculation, nausea and vomiting, choking on the diaphragm, asthma, seizures, vomiting blood, nosebleeds, and diarrhea
Sojae Semen Praeparatum	Fermented and processed mature seeds of soybean, a leguminous plant	Febrile fever, cold and heat, headache, irritability, chest tightness
Mume Fructus	The dried immature fruit of the Rosaceae plant plum	Long-term cough, deficiency heat and thirst, long-term malaria, long-term diarrhea, dysentery, bloody stool and urine, abdominal pain and vomiting caused by roundworms, hookworm disease, psoriasis
Dried Ginger	Dried rhizomes of Zingiber officinale Rosc, a plant in the ginger family	Heart and abdomen cold pain, vomiting and diarrhea, slight cold pulse in limbs, wind cold dampness obstruction, Yang deficiency vomiting, bleeding, and diarrhea
Cinnamomi Ramulus	The tender branches of cinnamon, a plant in the Lauraceae family	Wind cold exterior syndrome, soreness and pain in the shoulder and back limbs, chest obstruction, and amenorrhea
Chuanxiong Rhizoma	The rhizome of Ligusticum chuanxiong, a plant in the Umbelliferae family	Menstrual disorders, menstrual cramps, abdominal pain, chest and rib pain, swelling and pain from falls, headache, rheumatism and rheumatism
Plum blossom	Green Calyx Plum of Rosaceae	Depressed and upset, hepatogenous gastralgia, globus hystericus
Caulis Bambusae in Taenia	The middle layer scraped off from the outer skin of the stems of grasses such as bamboo and bamboo	Vomiting, hiccups, coughing and wheezing caused by heat, vomiting blood, bleeding, nausea, and seizures
Fennel	Dried and ripe fruit of the Umbelliferae plant *Foeniculum vulgare* Mill	cold hernia, Testicular prolapse, dysmenorrhea, lower abdominal cold pain, abdominal distension and pain, low appetite, vomiting and diarrhea, and testicular hydrocele
Dandelion	Rooted whole plant for the Asteraceae plant dandelion	Acute mastitis, lymphadenitis, acute conjunctivitis, cold fever, acute tonsillitis, acute bronchitis, gastritis, hepatitis, cholecystitis, urinary tract infection
Panax notoginseng	The root tuber of Panax notoginseng, a plant in the Panax genus of the Araliaceae family	Vomiting blood, coughing up blood, bleeding, rectal bleeding, bloody dysentery, collapse, postpartum dizziness, bruises, external bleeding, swelling and pain
Amomi fructus	Dried and ripe fruits of ginger plants such as *Amomum villosum* Lour or *Amomum longiligulare* T. L. Wu	Abdominal distension without hunger, spleen and stomach deficiency and cold, vomiting and diarrhea, pernicious vomiting, threatened abortion
Pericarpium Trichosanthes	For the Cucurbitaceae plant Dry and mature fruit peels of *Trichosanthes rosthonii* Harms or bilateral trichosanthes rosthonii	Cough caused by phlegmheat, pharyngeal pain, chest pain, vomiting blood, bleeding, consumptive thirst, constipation, abscess and swelling

In terms of outcomes, 11 studies reported overall clinical efficacy, 5 studies reported recurrence rate, 10 studies reported improvement of acid regurgitation, 9 studies reported improvement of heartburn, 8 studies reported improvement of esophageal chest pain, 5 studies reported FSSG score, 10 studies reported adverse events. The details are shown in Table 1.

### 3.2. Risk of bias evaluation

**Figure 2.**
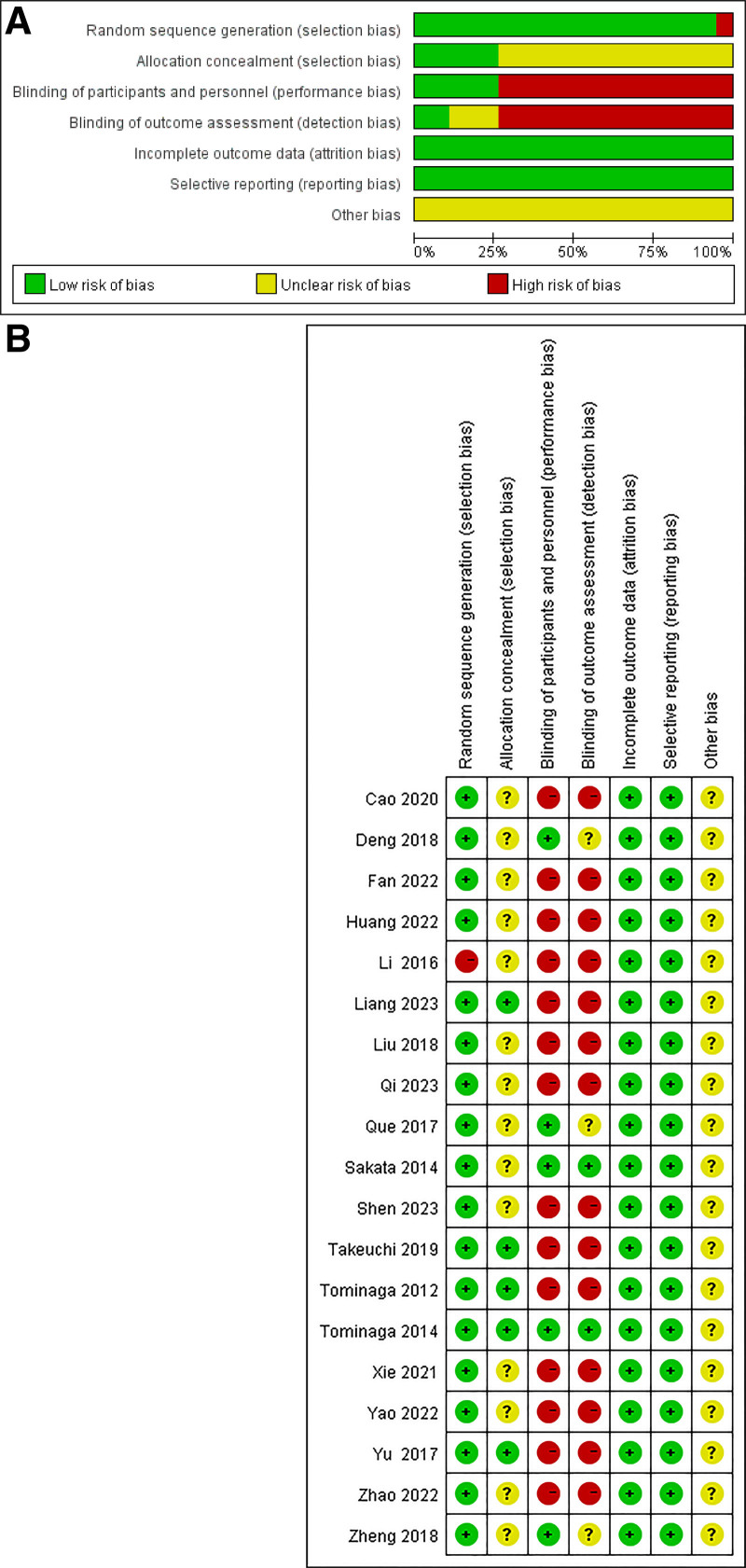
Risk of bias (A) summary and (B) graph.

The Cochrane Collaborative recommendation evaluation tool was used to conduct quality evaluation of the 19 included studies, as shown in Figure 2A,B. About 95.65% (18/19) of the studies were randomized controlled trials, of which 78.95% (15/19) described specific random assignment methods, such as random number tables or computer-generated randomization list, and the remaining 21.05% (4/19) only mentioned the word “randomization” without any explanation. About 21.05% (4/19) of the studies assigned allocation concealment through methods such as patient registration centers or sealed opaque envelopes, 78.95% (15/19) because of insufficient information about allocation concealment and were judged as of “unclear risk.” In terms of performance bias, 26.32% (5/19) of the studies used double-blind method, and the remaining 73.68% (14/19) of the studies did not use blind method. In terms of detection bias, only 10.53% (2/19) of the studies used blinding for the outcome evaluation, and 89.47% (17/19) of the studies did not have blinding or were not clear about blinding. All studies reported the completeness of outcome data, 47.37% (9/19) of the studies had no missing outcome data, and 52.63% (10/19) of the studies provided the number of dropout cases and detailed explanation of their causes. In addition, all studies had insufficient information about other risks and were therefore judged to be “unclear risk.”

### 3.3. Pairwise meta-analysis

#### 3.3.1. Overall clinical efficacy

**Figure 3.**
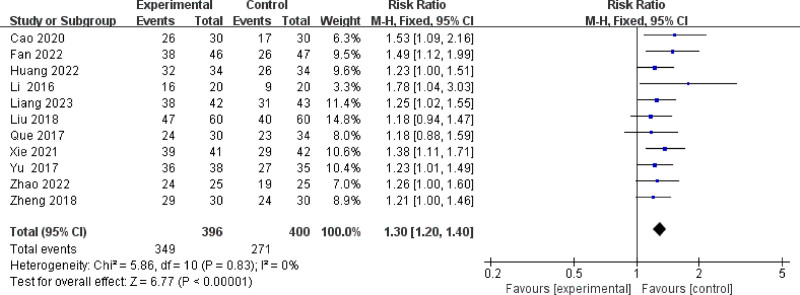
Overall clinical efficacy. CI = confidence interval.

11 studies (736 patients) were included in the meta-analysis of overall clinical efficacy, *P* = .83, *I*^2^ = 0%, which suggested that there was little heterogeneity among the studies, and a fixed-effect model was adopted. As shown in Figure 3, the total clinical effective rate of integrated Chinese and Western medicine was significantly better than that of conventional Western medicine (RR: 1.30; 95% CI: 1.20–1.40; *P* < .00001).

#### 3.3.2. Recurrence rate

**Figure 4.**
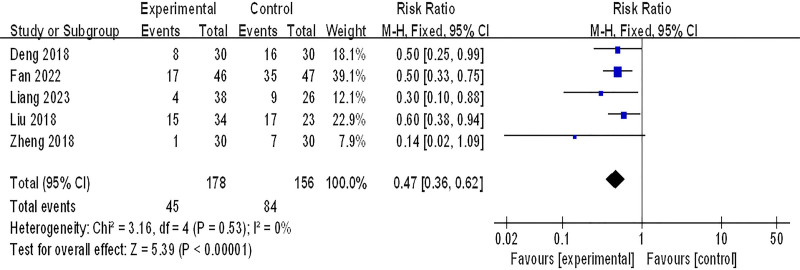
Recurrence rate. CI = confidence interval.

Five studies (334 patients) were included in the meta-analysis of recurrence rate, *P* = .53, *I*^2^ = 0%, which suggested that there was little heterogeneity among the studies, and a fixed-effect model was adopted. As shown in Figure 4, the recurrence rate of integrated Chinese and Western medicine was significantly lower than that of conventional Western medicine (RR: 0.47; 95% CI: 0.36–0.62; *P* < .00001).

#### 3.3.3. Improvement of acid regurgitation

**Figure 5.**
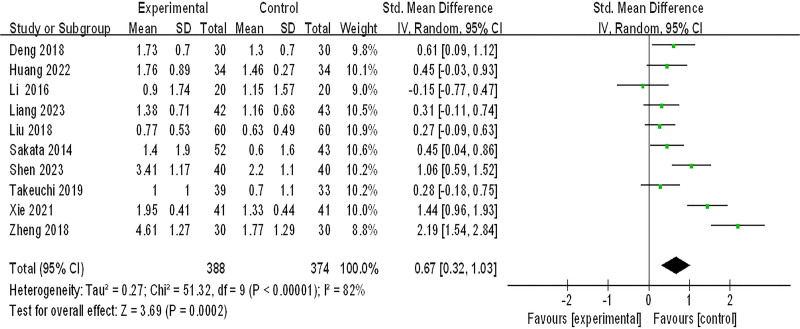
Improvement of acid regurgitation. CI = confidence interval, SD = standard deviation.

Ten studies (762 patients) were included in the meta-analysis of improvement of acid regurgitation, *P* < .00001, *I*^2^ = 82%, which suggested that there was great heterogeneity among the studies, and the sensitivity analysis showed no significant change in heterogeneity, and a random effects model was adopted. As shown in Figure 5, the effect of integrated Chinese and Western medicine treatment is significantly better than conventional Western medicine treatment in improving the acid regurgitation (SMD = 0.67, 95% CI: (0.32–1.03), *Z* = 3.69, *P* = .0002).

#### 3.3.4. Improvement of heartburn

**Figure 6.**
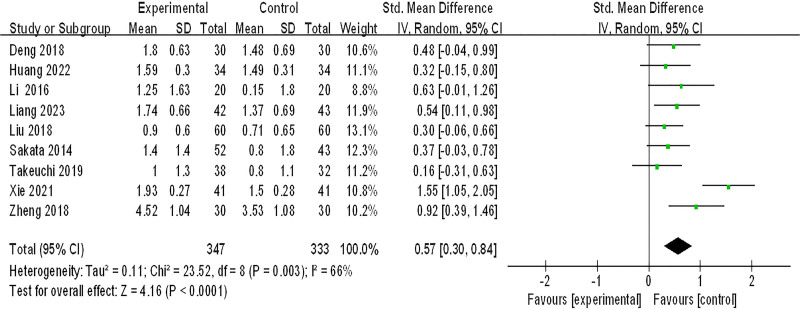
improvement of heartburn. CI = confidence interval, SD = standard deviation.

Nine studies (680 patients) were included in the meta-analysis of improvement of heartburn, *P* = .003, *I*^2^ = 66%, which suggested that there was great heterogeneity among the studies, and the sensitivity analysis showed no significant change in heterogeneity, and a random effects model was adopted. As shown in Figure 6, the effect of integrated Chinese and Western medicine treatment is significantly better than conventional Western medicine treatment in improving heartburn (SMD = 0.57, 95% CI (0.30, 0.84), *Z* = 4.16, *P* < .0001).

#### 3.3.5. Improvement of esophageal chest pain

**Figure 7.**
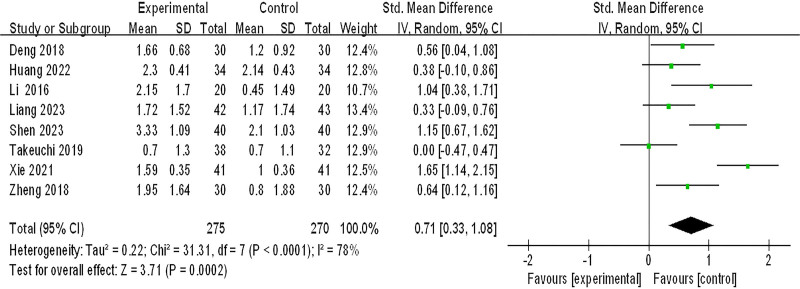
improvement of esophageal chest pain. CI = confidence interval, SD = standard deviation.

Eight studies (545 patients) were included in the meta-analysis of improvement of esophageal chest pain, *P* < .0001, *I*^2^ = 78%,which suggested that there was great heterogeneity among the studies, and the sensitivity analysis showed no significant change in heterogeneity, and a random effects model was adopted. As shown in Figure 7, the effect of integrated Chinese and Western medicine treatment is significantly better than conventional Western medicine treatment in improving the retrosternal pain (SMD = 0.71, 95% CI (0.33, 1.08), *Z* = 3.71, *P* = .0002).

#### 3.3.6. FSSG score

**Figure 8.**
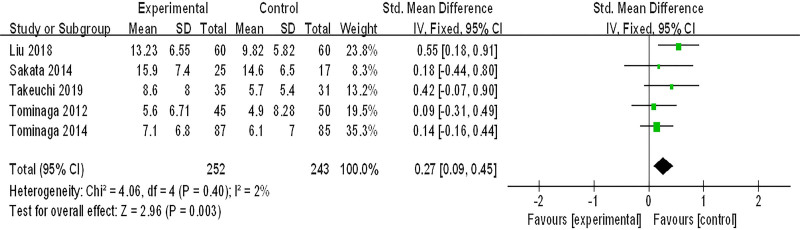
FSSG score. CI = confidence interval, FSSG = frequency scale for the symptoms of GERD, SD = standard deviation.

Five studies (495 patients) were included in the meta-analysis of improvement of FSSG score, *P* < .4, *I*^2^ = 2%, which suggested that there was little heterogeneity among the studies, and a fixed-effect model was adopted. As shown in Figure 8, the effect of integrated Chinese and Western medicine treatment is significantly better than conventional Western medicine treatment in improving FSSG score (SMD = 0.27, 95% CI (0.09, 0.45), *Z* = 4.16, *P* = .003).

#### 3.3.7. Adverse events

**Figure 9.**
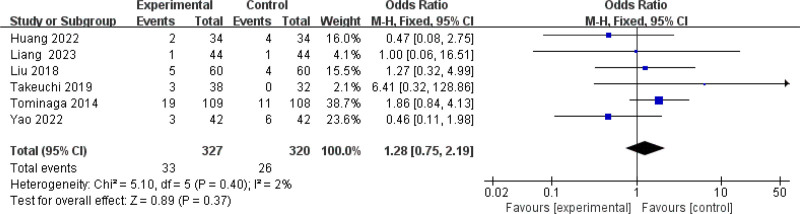
Adverse events. CI = confidence interval.

Ten studies (945 patients) were included in the meta-analysis of adverse events, *P* = .40, *I*^2^ = 2%, which suggested that there was little heterogeneity among the studies, and a fixed-effect model was adopted. Of these, the number of adverse events in both the experimental and control groups was 0 in 4 studies, and the results of the other 6 studies were not statistically significant. As shown in Figure 9, the combined results were not statistically significant (SMD = 1.28, 95% CI (0.75, 2.19), *Z* = 0.89, *P* = .37). This indicated that there was no statistically significant difference in the incidence of adverse events between the 2 groups. No treatment-related serious adverse events were reported in all studies, which were mild and resolved on their own or after discontinuation. Therefore, both treatment methods are relatively safe.

### 3.4. Network meta-analysis

#### 3.4.1. Network map

**Figure 10.**
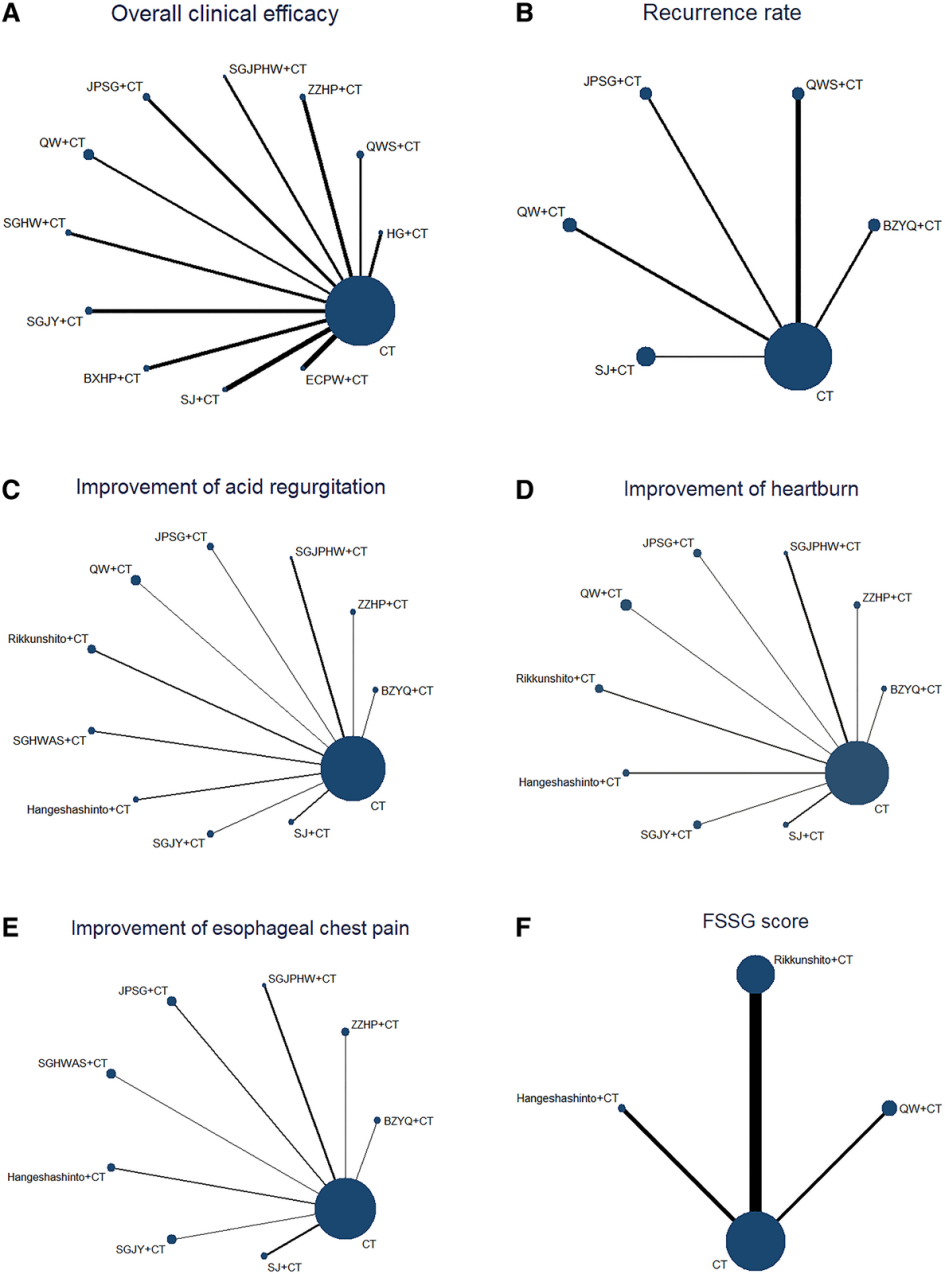
Network evidence diagram of overall clinical efficacy (A) recurrence rate, (B) acid regurgitation, (C) improvement of heartburn, and (d) esophageal chest pain. BXHP = Banxia Houpu Decoction, BZYQ = Buzhongyiqi Granules, CT = conventional Western medicine, ECPW = Erchen Pingwei Power, HG = Huagan Decoction, QW = Qingwei Granules, JPSG = Jianpi Shugan Decoction, QWS = Qingweishu Granules, SGHW = Shugan Hewei Decoction, SGHWAS = Shugan Hewei Anshen Prescription, SGJY = Shugan Jieyu Capsule, SGJPHW = Shugan Jianpi Hewei Decoction, SJ = Sanji Powders, ZZHP = Zhizi Houpo Decoction and Zhizi Douchi Decoction Combined with Wumei Pill.

Eleven studies reported overall clinical efficacy involving 11 CHMs, forming 11 direct comparisons; 5 studies reported recurrence rate involving 5 CHMs, forming 5 direct comparisons; 10 studies reported improvement of acid regurgitation involving 10 CHMs, forming 10 direct comparisons; 9 studies reported improvement of heartburn involving 9 CHMs, forming 9 direct comparisons; 8 studies reported improvement of esophageal chest pain involving 8 CHMs, forming 8 direct comparisons; 5 studies reported improvement of FSSG score involving 3 CHMs, forming 3 direct comparisons. As shown in Figure 10.

#### 3.4.2. Overall clinical efficacy

A total of 11 studies compared the overall clinical efficacy, involving 11 CHMs. The results of network analysis showed that the efficacy of HG + CT (OR = 4.97, 95% CI [1.39, 17.83]), QWS + CT (OR = 3.84, 95% CI [1.48, 9.97]), SGJPHW + CT (OR = 4.89, 95% CI [1.20, 19.94]), JPSG + CT (OR = 3.68, 95% CI [1.08, 12.54]), SGJY + CT (OR = 8.74, 95% CI [1.83, 41.78]), BXHP + CT (OR = 5.33, 95% CI [1.05, 27.16]) were superior to that of conventional Western medicine alone, and the difference was statistically significant (*P* < .05). As shown in Table 3.

**Table 3:** Network meta-analysis of overall clinical efficacy.

Intervention	OR [95% CI]											
	HG + CT	QWS + CT	ZZHP + CT	SGJPHW + CT	JPSG + CT	QW + CT	SGHW + CT	SGJY + CT	BXHP + CT	SJ + CT	ECPW + CT	CT
HG + CT	0											
QWS + CT	1.30 (0.26, 6.38)	0										
ZZHP + CT	1.01 (0.13, 8.03)	0.78 (0.12, 5.17)	0									
SGJPHW + CT	1.02 (0.15, 6.79)	0.78 (0.14, 4.29)	1.01 (0.12, 8.69)	0								
JPSG + CT	1.35 (0.23, 7.94)	1.04 (0.22, 4.94)	1.34 (0.17, 10.33)	1.33 (0.21, 8.59)	0							
QW + CT	2.75 (0.60, 12.52)	2.12 (0.60, 7.45)	2.72 (0.44, 16.91)	2.70 (0.53, 13.74)	2.03 (0.47, 8.88)	0						
SGHW + CT	2.60 (0.47, 14.47)	2.01 (0.45, 8.92)	2.57 (0.35, 18.94)	2.56 (0.42, 15.69)	1.92 (0.36, 10.31)	0.94 (0.23, 3.86)	0					
SGJY + CT	0.57 (0.08, 4.28)	0.44 (0.07, 2.74)	0.56 (0.06, 5.41)	0.56 (0.07, 4.58)	0.42 (0.06, 3.07)	0.21 (0.04, 1.21)	0.22 (0.03, 1.52)	0				
BXHP + CT	0.93 (0.12, 7.38)	0.72 (0.11, 4.75)	0.92 (0.09, 9.26)	0.92 (0.11, 7.88)	0.69 (0.09, 5.29)	0.34 (0.05, 2.09)	0.36 (0.05, 2.63)	1.64 (0.17, 15.67)	0			
SJ + CT	0.69 (0.05, 8.61)	0.53 (0.05, 5.74)	0.68 (0.04, 10.39)	0.67 (0.05, 9.06)	0.51 (0.04, 6.22)	0.25 (0.02, 2.57)	0.26 (0.02, 3.11)	1.21 (0.08, 17.71)	0.74 (0.05, 11.22)	0		
ECPW + CT	0.66 (0.05, 8.35)	0.51 (0.05, 5.57)	0.65 (0.04, 10.07)	0.65 (0.05, 8.79)	0.49 (0.04, 6.03)	0.24 (0.02, 2.49)	0.25 (0.02, 3.02)	1.15 (0.08, 17.16)	0.70 (0.05, 10.87)	0.96 (0.04, 21.26)	0	
CT	**4.97 (1.39, 17.83**)	**3.84 (1.48, 9.97**)	4.92 (0.96, 25.22)	**4.89 (1.20, 19.94**)	**3.68 (1.08, 12.54**)	1.81 (0.80, 4.09)	1.91 (0.61, 6.03)	**8.74 (1.83, 41.78**)	**5.33 (1.05, 27.16**)	7.25 (0.82, 64.45)	7.58 (0.84, 68.45)	0

Compare the improvement of outcome indicators between two different intervention measures, and highlight the statistically significant ones in bold.

BXHP = Banxia Houpu Decoction, CI = confidence interval, CT = conventional Western medicine, ECPW = Erchen Pingwei Power, HG = Huagan Decoction, JPSG = Jianpi Shugan Decoction, QW = Qingwei Granules, QWS = Qingweishu Granules, SGHW = Shugan Hewei Decoction, SGJPHW = Shugan Jianpi Hewei Decoction, SGJY = Shugan Jieyu Capsule, SJ = Sanji Powders, ZZHP = Zhizi Houpo Decoction and Zhizi Douchi Decoction Combined with Wumei Pill.

#### 3.4.3. Recurrence rate

A total of 5 studies compared the recurrence rate, involving 5 CHMs. The results of network analysis showed that the efficacy of QW + CT (OR = 0.32, 95% CI [0.11, 0.94]), SJ + CT (OR = 0.28, 95% CI [0.09, 0.88]), BZYQ + CT (OR = 0.11, 95% CI [0.01, 0.99]), JPSG + CT (OR = 0.22, 95% CI [0.06, 0.83]) and QWS + CT (OR = 0.20, 95% CI [0.08, 0.49]) were superior to that of conventional Western medicine alone, and the difference was statistically significant (*P* < .05). As shown in Table 4.

**Table 4:** Network meta-analysis of recurrence rate.

Intervention	OR [95% CI]					
	QW + CT	SJ + CT	BZYQ + CT	JPSG + CT	QWS + CT	CT
QW + CT	0					
SJ + CT	1.14 (0.24, 5.54)	0				
BZYQ + CT	2.81 (0.25, 31.59)	2.46 (0.21, 28.56)	0			
JPSG + CT	1.43 (0.26, 7.85)	1.25 (0.22, 7.19)	0.51 (0.04, 6.42)	0		
QWS + CT	1.58 (0.39, 6.41)	1.39 (0.32, 5.93)	0.56 (0.05, 5.86)	1.11 (0.23, 5.40)	0	
CT	**0.32 (0.11, 0.94**)	**0.28 (0.09, 0.88**)	**0.11 (0.01, 0.99**)	**0.22 (0.06, 0.83**)	**0.20 (0.08, 0.49**)	0

Compare the improvement of outcome indicators between two different intervention measures, and highlight the statistically significant ones in bold.

BZYQ = Buzhongyiqi Granules, CI = confidence interval, CT = conventional Western medicine, JPSG = Jianpi Shugan Decoction, QW = Qingwei Granules, QWS = Qingweishu Granules, SJ = Sanji Powders.

#### 3.4.4. Improvement of acid regurgitation

A total of 10 studies compared the improvement of acid regurgitation, involving 10 CHMs. The results of network analysis showed that the efficacy of BZYQ + CT (OR = 1.54, 95% CI [1.03, 2.29]), Rikkunshito + CT (OR = 2.23, 95% CI [1.10, 4.50]), SGHWAS + CT(OR = 3.35, 95% CI [2.04, 5.52]), SGJY + CT (OR = 1.86, 95% CI [1.55, 2.24]) and SJ + CT (OR = 17.12, 95% CI [8.96, 32.71]) were superior to that of conventional Western medicine alone, and the difference was statistically significant (*P* < .05). As shown in Table 5.

**Table 5:** Network meta-analysis of improvement of acid regurgitation.

Intervention	OR [95% CI]										
	BZYQ + CT	ZZHP + CT	SGJPHW + CT	JPSG + CT	QW + CT	Rikkunshito + CT	SGHWAS + CT	Hangeshashinto + CT	SGJY + CT	SJ + CT	CT
BZYQ + CT	0										
ZZHP + CT	1.14 (0.68, 1.90)	0									
SGJPHW + CT	1.97 (0.66, 5.95)	1.73 (0.59, 5.07)	0								
JPSG + CT	1.23 (0.75, 2.04)	1.08 (0.70, 1.67)	0.63 (0.21, 1.82)	0							
QW + CT	1.34 (0.86, 2.09)	1.17 (0.82, 1.69)	0.68 (0.24, 1.92)	1.08 (0.77, 1.53)	0						
Rikkunshito + CT	0.69 (0.31, 1.55)	0.61 (0.28, 1.31)	0.35 (0.10, 1.22)	0.56 (0.26, 1.20)	0.52 (0.25, 1.07)	0					
SGHWAS + CT	0.46 (0.24, 0.87)	0.40 (0.22, 0.72)	0.23 (0.07, 0.73)	0.37 (0.21, 0.66)	0.34 (0.20, 0.58)	0.66 (0.28, 1.57)	0				
Hangeshashinto + CT	1.14 (0.60, 2.15)	1.00 (0.56, 1.79)	0.58 (0.18, 1.80)	0.92 (0.52, 1.64)	0.85 (0.51, 1.44)	1.65 (0.70, 3.89)	2.48 (1.24, 4.99)	0			
SGJY + CT	0.83 (0.53, 1.29)	0.73 (0.51, 1.04)	0.42 (0.15, 1.19)	0.67 (0.47, 0.95)	0.62 (0.48, 0.80)	1.20 (0.58, 2.48)	1.80 (1.06, 3.07)	0.73 (0.43, 1.22)	0		
SJ + CT	0.09 (0.04, 0.19)	0.08 (0.04, 0.16)	0.05 (0.01, 0.15)	0.07 (0.04, 0.15)	0.07 (0.03, 0.13)	0.13 (0.05, 0.34)	0.20 (0.09, 0.44)	0.08 (0.04, 0.18)	0.11 (0.06, 0.21)	0	
CT	**1.54 (1.03, 2.29**)	1.35 (0.99, 1.85)	0.78 (0.28, 2.18)	1.25 (0.93, 1.67)	1.15 (0.96, 1.38)	**2.23 (1.10, 4.50**)	**3.35 (2.04, 5.52**)	1.35 (0.83, 2.20)	**1.86 (1.55, 2.24**)	**17.12 (8.96, 32.71**)	0

Compare the improvement of outcome indicators between two different intervention measures, and highlight the statistically significant ones in bold.

BZYQ = Buzhongyiqi Granules, CI = confidence interval, CT = conventional Western medicine, JPSG = Jianpi Shugan Decoction, QW = Qingwei Granules, SGHWAS = Shugan Hewei Anshen Prescription, SGJPHW = Shugan Jianpi Hewei Decoction, SGJY = Shugan Jieyu Capsule, SJ = Sanji Powders, ZZHP = Zhizi Houpo Decoction and Zhizi Douchi Decoction Combined with Wumei Pill.

#### 3.4.5. Improvement of heartburn

A total of 9 studies compared the improvement of heartburn, involving 9 CHMs. The results of network analysis showed that the efficacy of JPSG + CT (OR = 1.72, 95% CI [1.12, 2.65]), SGJY + CT (OR = 4.70, 95% CI [2.86, 7.73]) and SJ + CT (OR = 2.51, 95% CI [1.47, 4.29]) were superior to that of conventional Western medicine alone, and the difference was statistically significant (*P* < .05). As shown in Table 6.

**Table 6:** Network meta-analysis of Improvement of heartburn.

Intervention	OR [95% CI]									
	BZYQ + CT	ZZHP + CT	SGJPHW + CT	JPSG + CT	QW + CT	Rikkunshito + CT	Hangeshashinto + CT	SGJY + CT	SJ + CT	CT
BZYQ + CT	0									
ZZHP + CT	1.17 (0.58, 2.35)	0								
SGJPHW + CT	0.86 (0.38, 1.95)	0.74 (0.33, 1.64)	0							
JPSG + CT	0.94 (0.48, 1.84)	0.80 (0.42, 1.53)	1.09 (0.50, 2.35)	0						
QW + CT	1.19 (0.64, 2.23)	1.02 (0.56, 1.86)	1.39 (0.67, 2.88)	1.27 (0.72, 2.24)	0					
Rikkunshito + CT	1.11 (0.58, 2.14)	0.95 (0.51, 1.78)	1.29 (0.61, 2.75)	1.18 (0.65, 2.15)	0.93 (0.54, 1.60)	0				
Hangeshashinto + CT	1.37 (0.68, 2.75)	1.17 (0.60, 2.30)	1.59 (0.72, 3.51)	1.46 (0.77, 2.77)	1.15 (0.64, 2.08)	1.23 (0.66, 2.30)	0			
SGJY + CT	0.34 (0.17, 0.70)	0.29 (0.15, 0.59)	0.40 (0.18, 0.89)	0.37 (0.19, 0.71)	0.29 (0.16, 0.53)	0.31 (0.16, 0.59)	0.25 (0.13, 0.50)	0		
SJ + CT	0.64 (0.31, 1.35)	0.55 (0.27, 1.13)	0.75 (0.32, 1.71)	0.68 (0.34, 1.36)	0.54 (0.28, 1.03)	0.58 (0.30, 1.13)	0.47 (0.23, 0.96)	1.87 (0.90, 3.88)	0	
CT	1.61 (0.96, 2.70)	1.38 (0.86, 2.23)	1.87 (0.99, 3.54)	**1.72 (1.12, 2.65**)	1.35 (0.94, 1.94)	1.45 (0.97, 2.18)	1.18 (0.73, 1.89)	**4.70 (2.86, 7.73**)	**2.51 (1.47, 4.29**)	0

Compare the improvement of outcome indicators between two different intervention measures, and highlight the statistically significant ones in bold.

BZYQ = Buzhongyiqi Granules, CI = confidence interval, CT = conventional Western medicine, JPSG = Jianpi Shugan Decoction, QW = Qingwei Granules, SGJPHW = Shugan Jianpi Hewei Decoction, SGJY = Shugan Jieyu Capsule, SJ = Sanji Powders, ZZHP = Zhizi Houpo Decoction and Zhizi Douchi Decoction Combined with Wumei Pill.

#### 3.4.6. Improvement of esophageal chest pain

A total of 8 studies compared the improvement of esophageal chest pain, involving 8 CHMs. The results of network analysis showed that the efficacy of SGJPHW + CT (OR = 5.47, 95% CI [2.03, 14.74]), SGHWAS + CT (OR = 3.42, 95% CI [2.15, 5.45]), SGJY + CT (OR = 1.80, 95% CI [1.55, 2.11]) and SJ + CT (OR = 3.16, 95% CI [1.29, 7.71]) were superior to that of conventional Western medicine alone, and the difference was statistically significant (*P* < .05). As shown in Table 7.

**Table 7:** Network meta-analysis of Improvement of esophageal chest pain.

Intervention	OR [95% CI]								
	BZYQ + CT	ZZHP + CT	SGJPHW + CT	JPSG + CT	SGHWAS + CT	Hangeshashinto + CT	SGJY + CT	SJ + CT	CT
BZYQ + CT	0								
ZZHP + CT	1.35 (0.78, 2.34)	0							
SGJPHW + CT	0.29 (0.10, 0.88)	0.21 (0.08, 0.59)	0						
JPSG + CT	0.91 (0.39, 2.16)	0.68 (0.33, 1.39)	3.16 (0.94, 10.59)	0					
SGHWAS + CT	0.46 (0.23, 0.92)	0.34 (0.21, 0.57)	1.60 (0.54, 4.78)	0.51 (0.22, 1.17)	0				
Hangeshashinto + CT	1.58 (0.74, 3.38)	1.17 (0.65, 2.13)	5.47 (1.75, 17.10)	1.73 (0.71, 4.23)	3.42 (1.65, 7.10)	0			
SGJY + CT	0.88 (0.51, 1.50)	0.65 (0.51, 0.84)	3.03 (1.11, 8.27)	0.96 (0.47, 1.96)	1.90 (1.16, 3.09)	0.55 (0.31, 0.99)	0		
SJ + CT	0.50 (0.18, 1.40)	0.37 (0.15, 0.93)	1.73 (0.46, 6.58)	0.55 (0.18, 1.70)	1.08 (0.40, 2.96)	0.32 (0.11, 0.91)	0.57 (0.23, 1.41)	0	
CT	1.58 (0.96, 2.62)	1.17 (0.96, 1.43)	**5.47 (2.03, 14.74**)	1.73 (0.87, 3.47)	**3.42 (2.15, 5.45**)	1.00 (0.57, 1.75)	**1.80 (1.55, 2.11**)	**3.16 (1.29, 7.71**)	0

Compare the improvement of outcome indicators between two different intervention measures, and highlight the statistically significant ones in bold.

BZYQ = Buzhongyiqi Granules, CI = confidence interval, CT = conventional Western medicine, JPSG = Jianpi Shugan Decoction, SGHWAS = Shugan Hewei Anshen Prescription, SGJPHW = Shugan Jianpi Hewei Decoction, SGJY = Shugan Jieyu Capsule, SJ = Sanji Powders, ZZHP = Zhizi Houpo Decoction and Zhizi Douchi Decoction Combined with Wumei Pill.

#### 3.4.7. FSSG score

A total of 5 studies compared the improvement of FSSG score, involving 3 CHMs. The results of network analysis showed that the efficacy of QW + CT (OR = 30.23, 95% CI [3.30, 277.41]) was superior to that of conventional Western medicine alone, and the difference was statistically significant (*P* < .05). As shown in Table 8.

**Table 8:** Network meta-analysis of FSSG score.

Intervention	OR [95% CI]			
	QW + CT	Rikkunshito + CT	Hangeshashinto + CT	CT
QW + CT	0			
Rikkunshito + CT	11.58 (0.76, 176.06)	0		
Hangeshashinto + CT	1.66 (0.03, 85.76)	0.14 (0.00, 5.39)	0	
CT	**30.23 (3.30, 277.41**)	2.61 (0.54, 12.68)	18.18 (0.70, 474.02)	0

Compare the improvement of outcome indicators between two different intervention measures, and highlight the statistically significant ones in bold.

CI = confidence interval, FSSG = frequency scale for the symptoms of GERD, T = conventional Western medicine, QW = Qingwei Granules.

#### 3.4.8. SUCRA value

**Figure 11.**
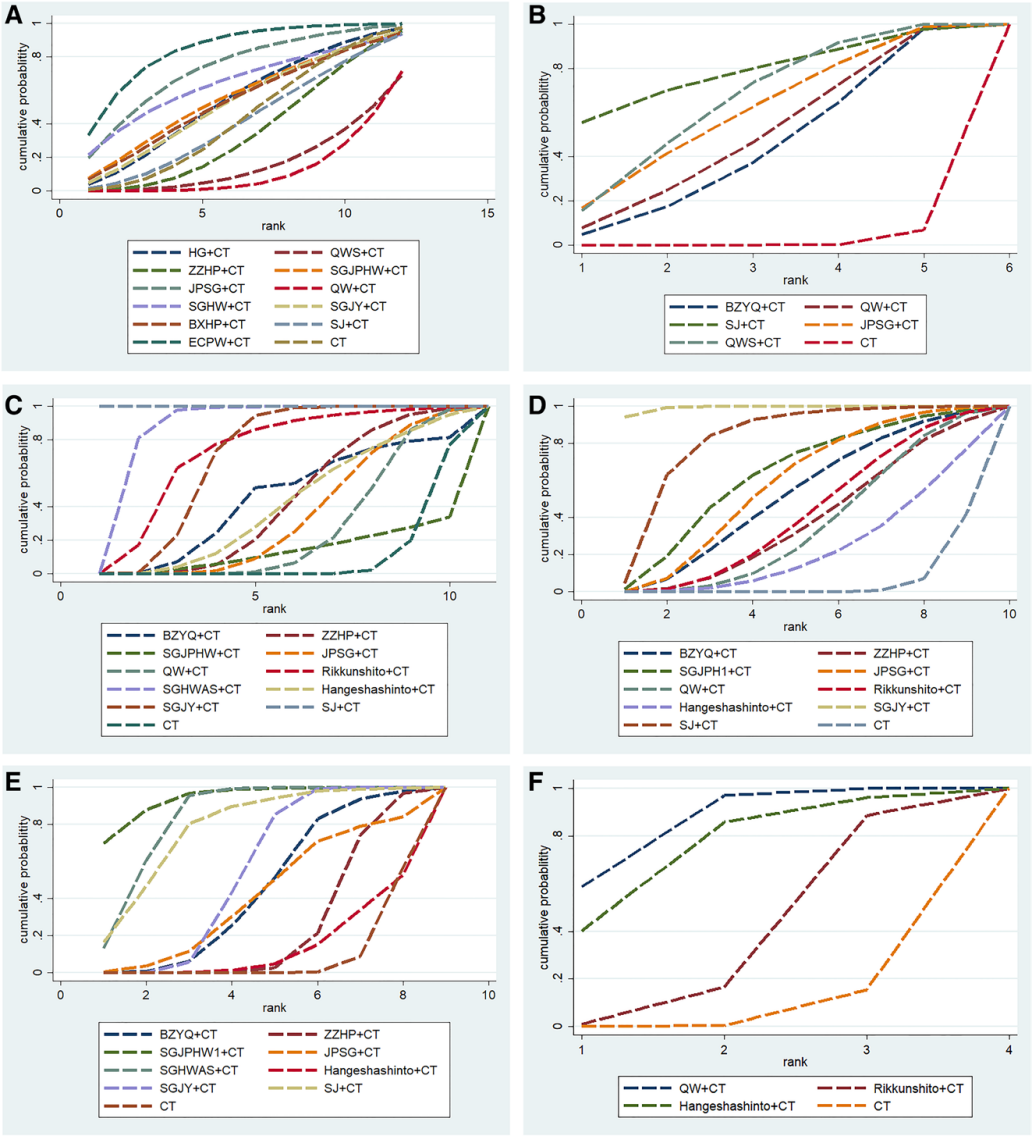
SUCRA curve ranking of different interventions to (A) overall clinical efficacy, (B) recurrence rate (C) improvement of acid regurgitation, (D) improvement of heartburn, (E) improvement of esophageal chest pain (F) FSSG score. BXHP = Banxia Houpu Decoction, BZYQ = Buzhongyiqi Granules, CT = conventional Western medicine, ECPW = Erchen Pingwei Power, FSSG = frequency scale for the symptoms of GERD, HG = Huagan Decoction, QW = Qingwei Granules, WS = Qingweishu Granules, SGHW = Shugan Hewei Decoction, SGHWAS = Shugan Hewei Anshen Prescription, SGJPHW = Shugan Jianpi Hewei Decoction, SGJY = Shugan Jieyu Capsule, SJ = Sanji Powders, JPSG = Jianpi Shugan Decoction, QWS = Qingweishu Granules, SUCRA = surface under the cumulative ranking curve, ZZHP = Zhizi Houpo Decoction and Zhizi Douchi Decoction Combined with Wumei Pill.

Cumulative probability ranking graph of each outcome indicator is shown in Figure 11. In terms of overall clinical efficacy, SGJY + CT is most likely to be the best intervention, and the specific order is: SGJY + CT (75.8%) > ECPW + CT (69.6%) > SJ + CT (67.3%) > BXHP + CT (61.4%) > HG + CT (59.3%) > ZZHP + CT (58.8%) > SGJPHW + CT (58.7%) > QWS + CT (50.3%) > JPSG + CT (48.1%) > SGHW + CT (25.3%) > QW + CT (22.0%) > CT (3.5%). In terms of recurrence rate, BZYQ + CT is most likely to be the best intervention, and the specific order is: BZYQ + CT (78.4%) > QWS + CT (65.3%) > JPSG + CT (60.4%) > SJ + CT (50.0%) > QW + CT (44.4%) >CT (1.4%). In terms of improvement of acid regurgitation, SJ + CT is most likely to be the best intervention, and the specific order is: SJ + CT (100.0%) > SGHWAS + CT (87.9%) > Rikkunshito + CT (72.6%) > SGJY + CT (69.1%) > BZYQ + CT (53.0%) > ZZHP + CT (42.3%) > Hangeshashinto + CT (40.9%) >JPSG + CT (34.4%) > QW + CT (26.5%) > SGJPHW + CT (13.4%) > CT (10.0%). In terms of improvement of heartburn, SGJY + CT is most likely to be the best intervention, and the specific order is: SGJY + CT (99.3%) > SJ + CT (82.0%) > SGJPHW + CT (63.2%) > JPSG + CT (58.3%) > BZYQ + CT (52.1%) > Rikkunshito + CT (42.1%) > ZZHP + CT (38.4%) > QW + CT (35.8%) > Hangeshashinto + CT (23.4%) > CT (5.5%). In terms of esophageal chest pain, SGJPHW + CT is most likely to be the best intervention, and the specific order is: SGJPHW + CT (94.1%) > SGHWAS + CT (83.7%) > SJ + CT (78.1%) > SGJY + CT (54.2%) > JPSG + CT (49.0%) > BZYQ + CT (44.8%) > ZZHP + CT (24.4%) > Hangeshashinto + CT (13.5%) > CT (8.2%). In terms of FSSG score, QW + CT is most likely to be the best intervention, and the specific order is: QW + CT (85.2%) > Hangeshashinto + CT (74.0%) > Rikkunshito + CT (35.4%) > CT (5.3%).

### 3.5. Inconsistency testing

The network evidence graph of the 6 outcome indicators shows that no closedloop has been formed, and no inconsistency test will be conducted.

### 3.6. Publication bias

**Figure 12.**
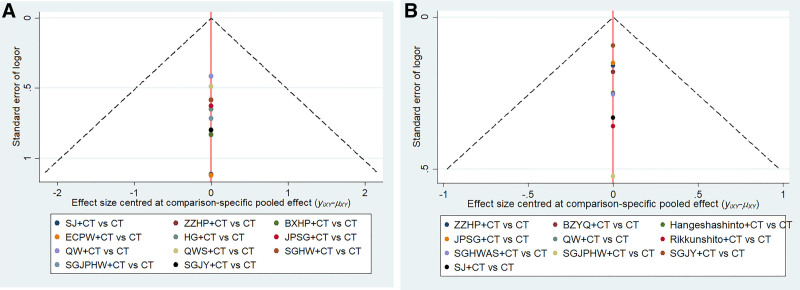
Comparison-corrected funnel plot of (A) overall clinical efficacy (B) improvement of acid regurgitation. BXHP = Banxia Houpu Decoction, BZYQ = Buzhongyiqi Granules, CT = conventional Western medicine, ECPW = Erchen Pingwei Power, HG = Huagan Decoction, JPSG = Jianpi Shugan Decoction, QW = Qingwei Granules, QWS = Qingweishu Granules, SGHW = Shugan Hewei Decoction, SGHWAS = Shugan Hewei Anshen Prescription, SGJPHW = Shugan Jianpi Hewei Decoction, SGJY = Shugan Jieyu Capsule, SJ = Sanji Powders, ZZHP = Zhizi Houpo Decoction and Zhizi Douchi Decoction Combined with Wumei Pill.

An evaluation of publication bias was conducted on overall clinical efficacy and improvement of acid regurgitation, as shown in Figure 12. The funnel plot is basically symmetrical, indicating no significant publication bias.

## 4. Discussion

### 4.1. Summary of findings

Pairwise meta-analysis showed that compared with conventional Western medicine treatment, CHM combined with conventional Western medicine treatment is more effective in overall clinical efficacy, reducing recurrence rate, improvement of acid regurgitation, improvement of heartburn, improvement of esophageal chest pain and FSSG score. Network meta-analysis show that in terms of overall clinical efficacy, SGJY + CT has the best effect; In terms of recurrence rate, BZYQ + CT has the best effect; In terms of improvement of acid regurgitation, SJ + CT has the best effect; In terms of improvement of heartburn, SGJY + CT has the best effect; In terms of improvement of esophageal chest pain, SGJPHW + CT has the best effect; In terms of FSSG score, QW + CT has the best effect. No serious adverse events were reported in all studies.

### 4.2. Advantages of combining CHM treatment

The pathophysiologic factors contributing to rGERD are multifarious, including different anatomical and functional abnormalities such as weakened antireflux barrier function, decreased esophageal clearance, delayed gastric emptying, esophageal hypersensitivity and hypervigilance.^[5]^ PPIs is the preferred treatment for rGERD, mainly by inhibiting stomach acid secretion to relieve symptoms. However, studies have shown that the persistence of symptoms in many patients is related to nonacid reflux, mucosal microscopic damage, psychological factors, etc.^[33]^ Therefore, many patients in clinical practice have not achieved ideal treatment effects even after optimizing the PPIs treatment plan. Although prokinetic drugs are commonly used in Asian countries to supplement the therapeutic effects of PPIs, a meta-analysis showed that this combination therapy has no significant effect on symptom improvement in GERD.^[34]^

However, many studies have shown that CHM may have a positive effect on multifarious pathologic mechanisms of rGERD. In terms of gastric emptying function, clinical studies have shown that Rikkunshito can improve gastric emptying delay.^[35]^ Animal studies have shown Hangeshashinto can improve delayed gastric emptying by modulating interstitial cells of Cajal and the smooth muscle cells in stomach.^[36]^ In terms of improving esophageal function, a clinical trial has shown that the Shugan Jianpi Hewei formula may achieve therapeutic effects on rGERD by reducing PAR2 expression, thereby reducing esophageal hypersensitivity.^[37]^ Network pharmacological studies showed that Huagan Decoction played an anti-inflammatory role by inhibiting pro-inflammatory cytokinesand regulating p38MAPK/NF-κB signaling pathway, thus restoring esophagus tissue homeostasis during inflammation.^[10]^ In addition, pharmacological studies have found that CHM such as Eugenia caryophyllata Thunb, Panax notaginseng, Bletilla striata, Cynchum paniculatum and Flos Inulae have anti-inflammatory, immunomodulatory, and mucosal repair effects.^[38–41]^ In terms of improving patients’ anxiety and depression, a meta-analysis is suggests that up to 33.3% of patients with GERD may experience depressive symptoms, and there may be a bidirectional causal relationship between the 2.^[42]^ Therefore, it is crucial to include antidepressants in the treatment of rRGERD. A study has shown that Shugan Jieyu Capsules can treat depression by regulating targets and signaling pathways through the active ingredients it contains.^[43]^ Another study has shown that Yueju Pill has a significant antidepressant effect.^[44]^ In our analysis, 10/19 (52.63%) of the studies mentioned “Shugan” treatment in the treatment principles of the experimental group. About 2/19 (10.53%) study mentioned an improvement in the Hamilton Anxiety Scale score in outcome indicators,^[21,30]^ and 1/19 (5.2%) study mentioned an improvement in the Hamilton depression scale score in outcome indicators,^[30]^ all of which indicate that the experimental group performed more significantly in improving the symptoms of anxiety and depression. In terms of cancer prevention, long-term gastroesophageal reflux can lead to recurrent esophageal cell proliferation and cause precancerous lesions, such as Barrett’s esophagus.^[45]^ Animal studies have shown that Hangeshashinto can reduce the incidence rate of cancer in surgical induced gastroduodenal-esophageal reflux rodent models.^[46]^ Pharmacological studies have shown that herbs such as Eugenia caryophyllata Thunb, Bletilla striata, Flos Inulae and Cynchum paniculatum have significant anticancer effects.^[38,40,41,47]^

In summary, the intervention of CHM in rGERD has the characteristics of multiple targets and pathways, which is worth further research to compensate for some deficiencies of PPIs treatment and provide new methods for the treatment of rGERD.

### 4.3. Limitations

Limitations of this study: the included studies were only conducted in China and Japan, lacking comprehensive evaluation of patient data from different races and regions; the quality of included studies needs to be improved, with only 21.05% of the studies used assignment hiding, only 26.32% of the studies used double-blind method, only 10.53% of the studies used blinding for the outcome evaluation, which may lead to bias in selection, implementation and measurement; the objective outcome indicators included in the study are not comprehensive, such as scores of gastroscopy evaluation, 24-hour PH monitoring of the esophagus and esophageal pressure measurement; most studies have not reported follow-up results and lack attention to the long-term efficacy of drugs and patient prognosis.

## 5. Conclusions

Despite its limitations, this is the first review to use network meta-analysis to combine CHM with conventional Western medicine for the treatment of rGERD. The results indicate that CHM has a positive adjuvant therapeutic effect on rGERD. However, more large sample, multicenter and high-quality studies are needed to improve the existing evidence system.

## Author contributions

**Conceptualization:** Ruiting Zhang.

**Data curation:** Ruiting Zhang, Zhenyu Yang, Xiangbin Pan, Yingzhe Liu, Qiusi Huang.

**Formal analysis:** Ruiting Zhang.

**Investigation:** Ruiting Zhang, Zhenyu Yang.

**Methodology:** Ruiting Zhang, Zhenyu Yang, Xiangbin Pan, Yingzhe Liu, Qiusi Huang, Zexi Song, Qianqian Yao, Dongxia Li, Yuan Zhang.

**Project administration:** Ruiting Zhang.

**Supervision:** Ruiting Zhang.

**Visualization:** Zexi Song, Qianqian Yao, Dongxia Li, Yuan Zhang.

**Writing – original draft:** Ruiting Zhang, Zhenyu Yang.

**Writing – review & editing:** Ruiting Zhang, Zhenyu Yang.
